# Supply-side factors influencing informal payment for healthcare services in Tanzania

**DOI:** 10.1093/heapol/czab034

**Published:** 2021-05-21

**Authors:** Peter Binyaruka, Dina Balabanova, Martin McKee, Eleanor Hutchinson, Antonio Andreoni, Mary Ramesh, Blake Angell, Ntuli A Kapologwe, Masuma Mamdani

**Affiliations:** Department of Health System, Impact Evaluation and Policy, Ifakara Health Institute, PO Box 78373, Dar es Salaam, Tanzania; Department of Global Health and Development, London School of Hygiene and Tropical Medicine, 15-17 Tavistock Place, London WC1H 9SH, UK; Department of Global Health and Development, London School of Hygiene and Tropical Medicine, 15-17 Tavistock Place, London WC1H 9SH, UK; Department of Global Health and Development, London School of Hygiene and Tropical Medicine, 15-17 Tavistock Place, London WC1H 9SH, UK; UCL Institute for Innovation and Public Purpose, 11 Montague Street, London WC1B 5BP, UK; Department of Health System, Impact Evaluation and Policy, Ifakara Health Institute, PO Box 78373, Dar es Salaam, Tanzania; The George Institute for Global Health, University of New South Wales Sydney, Sydney, Australia; UCL Institute for Global Health, 30 Guilford Street, London WC1N 1EH, UK; President’s Office—Regional Administration and Local Government (PO-RALG), Dodoma, Tanzania; Department of Health System, Impact Evaluation and Policy, Ifakara Health Institute, PO Box 78373, Dar es Salaam, Tanzania

**Keywords:** Informal payment, corruption, health financing, health providers, Tanzania

## Abstract

Informal payments for healthcare are widespread in sub-Saharan Africa. They are often regressive, potentially limiting access to quality healthcare, particularly for the most vulnerable, and can have catastrophic consequences for households. Yet there is little empirical research that uses theory-driven hypotheses to explore what influences informal payments and, especially, from health workers’ perspectives. Consequently, we have explored the characteristics of health workers and facilities influencing informal payments in Tanzania, examining two hypotheses: health workers with power and position in the system are more likely to receive informal payments, and transparency and accountability measures can be bypassed by those who can game the system. We conducted a cross-sectional survey of 432 health workers from 42 public health facilities (hospitals and health centres) in 12 district councils from Pwani and Dar es Salam regions in Tanzania. Our dependent variable was whether the health worker has ever asked for or been given informal payments or bribes, while explanatory variables were measured at the individual and facility level. Given the hierarchical structure of the data, we used a multilevel mixed-effect logistic regression to explore the determinants. Twenty-seven percent of 432 health workers ever engaged in informal payment. This was more common amongst younger (<35 years) health workers and those higher in the hierarchy (specialists and heads of departments). Those receiving entitlements and benefits in a timely manner and who were subject to continued supervision were significantly less likely to receive informal payments. The likelihood of engaging in informal payments varied among health workers, consistent with our first hypothesis, but evidence on the second hypothesis remains mixed. Thus, policy responses should address both individual and system-level factors, including ensuring adequate and progressive health sector financing, better and timely remuneration of frontline public health providers, and enhanced governance and supervision.

## Introduction

Out-of-pocket payments (OOPs) for health care are a barrier to achieving Universal Health Coverage (WHO, 2010) but remain widespread in many low- and middle-income countries (LMICs), despite being inequitable, often regressive, and inefficient as a financing mechanism (Asante *et al.*, 2016). Many countries are working to extend and strengthen prepayment mechanisms (e.g. general taxation and health insurance) to ensure financial protection (WHO, 2010; Kutzin, 2013). However, they have often focused on formal OOP, giving less attention to informal payments (Hutchinson *et al.*, 2019). Informal payments include payments to individual healthcare workers or their facilities, in kind or in cash, made outside official payment channels, including payments for drugs/supplies that should be covered by the health system (Gaal *et al.*, 2006a; Lewis, 2007). They are encouraged by characteristics of clinical encounters, where health workers can exploit information asymmetry, uncertainty and power imbalance (Ensor and Witter, 2001; Savedoff, 2007; Vian, 2008) and the broader context (Schaaf and Topp, 2019).

The existing literature identifies three consequences of informal payments. First, they can create barriers to care (Ensor and Cooper, 2004; O'Donnell, 2007), increasing the risk of catastrophic expenditure (WHO, 2010) and undermining confidence in public institutions (Clausen *et al.*, 2011; Habibov, 2016). Second, they may respond to a dysfunctional system (Méon and Weill, 2010), overcoming inefficiencies or bureaucratic obstacles to service delivery (Leff, 1964; Gaal and McKee, 2004) or motivating providers to stay at their post, supplementing their low salaries (Gaal and McKee, 2004; Lewis, 2007; Mæstad and Mwisongo, 2011). They may even redistribute resources to the poor, when health workers only seek them from those who can pay (Ensor and Savelyeva, 1998) and can encourage public facilities to compete with private ones (Rose, 1998). Third, informal payments can represent cultural norms of gratitude (Smith, 2008; Truex, 2011; Lee and Guven, 2013), with material consequences only if they are large or frequent (Gaal *et al.*, 2006a).

Much research on informal payments is from Central and Eastern Europe (e.g. Thompson and Witter, 2000; Balabanova and McKee, 2002; Ensor, 2004; Gaal *et al.*, 2006b; Dabalen and Wane, 2008; Horodnic *et al.*, 2018), or Asia (e.g. Nahar and Costello, 1998; Barber *et al.*, 2004; Meskarpour Amiri *et al.*, 2019), with less in South America (e.g. Gatti *et al.*, 2003) and Africa (e.g. McPake *et al.*, 1999; Mæstad and Mwisongo, 2011; Kankeu and Ventelou, 2016). Several factors encourage persistence of informal payments, both demand-side (e.g. patients and community characteristics) and supply-side factors (e.g. health system and providers’ characteristics). However, most studies on the determinants of informal payment have focused on users’ (demand-side factors) rather than providers’ perspectives (supply-side factors) (Dabalen and Wane, 2008; Kankeu *et al.*, 2016); and few offer a theory-informed interpretation.

Here, we apply a novel lens to the analysis of informal payments in Tanzania, drawing on political settlement theory (Behuria, 2017; Khan, 2018). This focuses on the distribution of power among different actors in each sector-country context. It does so by looking at the capabilities of relevant actors (and their clientelist networks), and how these capabilities (and networks) allow them to mobilize resources and extract potential rents from existing policies and institutions (for a review of alternative political settlement approaches, see Behuria, 2017). This theory has been developed within the Anti-Corruption Evidence (ACE) research programme that includes Nigeria and Bangladesh along with Tanzania, the focus of this article (Khan *et al.*, 2019). For brevity, we refer to this as the ACE approach. It recognizes two key issues arising where formal rules are weakly enforced and widely violated by powerful groups. The first is the importance of disentangling the distribution of organizational power in each sector—its ‘political settlement’—and mapping the incentive structures arising from opportunities for rent-capture and vulnerability to corruption. Second, it hypothesizes that traditional top-down anti-corruption strategies—such as transparency and accountability measures—will be effective ‘only if’ they are complemented with bottom-up strategies, taking into account the sector-specific political settlement. If particular anti-corruption strategies are aligned with the interests of enough players who have sufficient bargaining power in a sector, and take into account differences in incentives and actors’ conditions, formal rules are more likely to be enforced over time.

In the health sector, at facility level in the privacy of the consultation room, health workers have power to provide or withhold services. However, some have more power than others, hence more or less scope to exploit corruption vulnerabilities and bypass formal enforcement mechanisms. Moreover, in their decisions, health workers are influenced by different working conditions and considerations. This heterogeneity matters not only for understanding drivers of informal payments, but also to design tailored anti-corruption responses and improve outcomes in the sector.

KEY MESSAGES

- Informal payments are widespread in Africa, yet there is little empirical research.
- In Tanzania, 27% of 432 health workers engaged in informal payments.
- Payments are less common where there is regular remuneration and supportive supervision.
- Existing transparency and accountability mechanisms are not associated with fewer payments.
- Anti-corruption approaches need to address both individual and system-level factors.

Drawing on our theoretical approach, we hypothesize that: (1) a particular subset of providers who have formal (and potentially informal) power and position within the health system may be more likely to obtain informal payments; (2) transparency and accountability measures such as electronic billing and public information are likely to be bypassed by those who can game the system. To test these hypotheses, we examine characteristics of health workers and health facilities associated with informal payments in Tanzania, where they are common and take many forms (Kruk *et al.*, 2008; Stringhini *et al.*, 2009; Mæstad and Mwisongo, 2011; Lindkvist, 2013). The first hypothesis has some support in the literature, mainly from outside Africa, but there is less related to the second one. The theoretical lenses we adopted in this study allows a more nuanced understanding of power distribution—both powers associated with formal status in the health facility, but also informal relationships. They also account for situations where powerful individuals can game the system and evade transparency and accountability rules while extracting large rents through informal payments.

## Study setting

This study took place in Tanzania, a lower middle-income country in East Africa with an estimated population of around 56 million people in 2016 (NBS, 2013). Tanzania is divided into 31 regions and most (70%) inhabitants reside in rural areas. Most health facilities (70%) are government owned. The public health system is organized hierarchically, with dispensaries, health centres and district hospitals providing primary healthcare; while regional referral, zonal and national hospitals provide secondary and tertiary care. The health system is highly decentralized and local providers have autonomy to plan and manage resources as their funds are received through direct health facility financing approach (Frumence *et al.*, 2013; Kapologwe *et al.*, 2019).

The Tanzanian health system is facing systemic challenges, including shortages and maldistribution of medicines and supplies (Wales *et al.*, 2014) and of health workers, with specialists concentrated in urban settings (Afnan-Holmes *et al.*, 2015). Health workers are paid monthly salaries, but these are widely perceived as insufficient to live on. Some health workers are eligible to receive certain allowances (extra duty, on-call, etc.) although these are often unpredictable and paid with frequent delays. These characteristics reflect inadequate health financing. For instance, in 2018/19, about 9% of Tanzanian government expenditure was allocated to health—below the Abuja declaration target of 15% (MOHCDGEC, 2019a).

The health financing system is highly fragmented. In 2015/16, for example, the healthcare budget came from general taxation (34%), donor support (36%), OOPs (22%) and health insurance contributions (8%) (MOHCDGEC, 2019b). In 2019, about 32% of Tanzanians were covered by health insurance, with 8% (public servants) in the National Health Insurance Fund, 23% (informal workers) by the Community Health Fund and 1% with private insurance (MOHCDGEC, 2019a). However, a majority, and especially informal workers, pay out-of-pocket. The poor and vulnerable groups (e.g. pregnant women, children and elders) should receive a fee-exemption and waiver in Tanzania, but these policies are weakly enforced and exempted people continue to pay out-of-pocket (Kruk *et al.*, 2008; Maluka, 2013).

## Materials and methods

### Data source

We conducted a cross-section survey of public health workers in eleven districts, five from the Dar es Salaam region and six from Pwani region. The two regions represent urban and rural/peri urban settings in Tanzania. The survey was undertaken from July to August 2019 across 42 health facilities. We included all public hospitals (*n* = 14, 33.3%) and health centres (*n* = 28, 66.6%) in two regions, excluding only the national hospital, military and specialized hospitals. We drew a convenience sample of health workers from those present on the day, selected purposively to include individuals in different departments and levels of seniority. We included a minimum of five medical staff at each facility. The final sample comprised 432 health workers from all 42 facilities (Table 1). One hundred and eighty-eight (43.5%) worked in hospitals and 244 (56.5%) in health centres. Trained enumerators collected data using a structured questionnaire loaded on tablet devices, and the interviews were undertaken in Swahili.

**Table 1:** Sample characteristics

	Number of healthcare workers	%
Region	(*n* = 432)	
Dar es Salaam	194	44.9
Pwani	238	55.1
Facility level of care	(*n* = 432)	
Hospitals	188	43.5
Health centres	244	56.5

### Outcome and explanatory variables

Our outcome of interest was informal payment, coded as 1 if a health worker has ever asked/given informal payment or bribe at the workplace, and 0 otherwise. This variable was measured in a survey through the following question: ‘Have you ever asked for/been given informal payment/bribe from clients?’

Given the hierarchical structure of the data, our explanatory variables were considered at two levels: individual- and facility. These two levels reflect our two hypotheses of providers’ power/position and availability of governance structure at facility, respectively. The ‘individual/health worker characteristics’ included: age, gender, health worker cadre, marital status, position at facility (whether in-charge or not), work experience in years and whether has any supplementary job for private income. Other variables such as education, qualification and salary, were dropped to avoid multicollinearity as they significantly correlated with medical/provider cadre (e.g. specialist, clinician or nurses) (a relatively important factor to inform policy interventions).

The ‘facility-level characteristics’ (also referred to as contextual/supply-side factors) included the following dummy variables: availability of supervision/oversight throughout, availability of electronic mode of payment, availability of an accountant, availability of a noticeboard to display fee structure and service offered, availability of health facility governing committee (HFGC) and level of care (i.e. hospital and health centre). Additional explanatory factors were continuous variables, rating various aspects of the working environment, such as availability of medical commodities, facility infrastructure condition, staffing level and condition regarding provision of entitlement and benefits, on a scale of 1 to 10.

## Analysis

Since individual health workers were nested within facilities, a multilevel modelling technique was preferred to account for the hierarchical structure of the data (Hox *et al.*, 2005; Rabe-Hesketh and Skrondal, 2008). Multilevel modelling estimates both individual and group-level effects simultaneously. We further assumed potential variation between facilities to support random effects estimation through mixed-effects model.

We therefore started by estimating a multilevel mixed-effect logistic regression. Prior to mixed-effects model, we estimated an empty/random intercept model to test through a likelihood test whether random intercept variations are present. The likelihood ratio test for the intercept only model was significant (*P* = 0.0177), indicating a significant improvement in fit with random intercepts compared to a standard logistic model. Thereafter, we estimated a mixed-effects model with multiple predictors (at individual and facility level), and obtained a likelihood ratio test with insignificant *P*-value of 0.3734. This indicated that the mixed-effects model does not represent a significant improvement in fit compared to a standard logistic model (Hox *et al.*, 2005). We similarly estimated the intraclass correlation (ICC) after the mixed-effects model, and obtained a value of 0.0156. Since the ICC is smaller approaching zero, there is evidence of limited between-facility variation as compared to within-facility variation. This support the decision not to use random effects but rather use a standard logistic model with clustering (Heck *et al.*, 2013). Based on prior results, we decided to estimate the significant determinants of engaging in informal payments through a standard logistic model while clustering at the facility level as follows.

(1) $${\rm{Logit}}\left\{ {pr\left( {{Y_{ij}}} \right)} \right\}{\rm{}} = {\rm{\alpha }} + {\rm{}}{\beta _1}{\rm{}}{X_{ij}}{\rm{}} + {\rm{}}{\beta _2}{Z_j}{\rm{}} + {\rm{}}{\varepsilon _{ij}}$$

Where *i* and *j* are individual health worker and health facility, respectively. ${Y_{ij}}$ is a binary outcome of interest (whether engaged in informal payment); ${X_{ij}}$ included individual health worker covariates, ${Z_{i}}$ included health facility level covariates, and ${\varepsilon _{ij}}$ is the individual error term. All analyses were performed in STATA 16.

## Results

### Descriptive statistics

Of 432 health workers surveyed, the majority were female (64%), nurses and midwives (39%), married (71%), subordinate to those in charge of units (54%) and did not have supplementary job(s) (61%) (Table 2). They averaged 7 years of experience at their working station. One hundred and seventeen (27.1%) reported engaging in informal payment by either asking for or receiving one at their workplace. Turning to the facilities where they worked, a few have continuous supervision (40%) and an electronic mode of payment (26%). However, most facilities have an accountant (91%), a noticeboard showing payment procedures and services offered (93%), and an HFGC (58%) (Table 2). Average ratings of the condition of the working environment (on a 1–10 scale, with 10 best) were around 6.6 for availability of medical commodities and infrastructure condition, while staffing levels and provision of entitlement and benefits were around 4.3.

**Table 2:** Description of providers’ characteristics and their probability of engaging in informal payment (*n* = 432)

		Sample		Engaging in informal payment (*n* = 117, 27.1%)	
Variables	Description	*n*	%	*n*	%
Panel A: Individual factors					
Age	20–34 years	154	35.7%	53	34.4%
	35–44 years	135	31.3%	32	23.7%
	45–60 years	143	33.1%	32	22.4%
Gender	Male	157	36.3%	42	26.7%
	Female	275	63.7%	75	27.3%
Medical cadre	Medical specialist	31	7.2%	12	38.7%
	Medical officer and Clinical officer	117	27.1%	35	29.9%
	Nurse and Midwives	167	38.7%	46	27.5%
	Others (e.g. paramedics)	117	27.1%	24	20.5%
Marital status	Married	306	70.8%	84	27.5%
	Not married	126	29.2%	33	26.2%
Position at the facility level	In-charge of the department/unit	199	46.1%	63	31.7%
	Not in-charge	233	53.9%	54	23.2%
Experience at the facility level	Number of years at a facility (SD)	432	6.9 (7.5)		
Supplementary job	Has any supplementary job to earn income	168	38.9%	49	29.2%
	No supplementary job	264	61.1%	68	25.8%
Panel B: Facility factors					
Supervision throughout	Facility with supervision throughout	171	39.6%	29	16.9%
	No	261	60.4%	88	33.7%
Electronic mode of payment	Yes—facility with electronic payment	114	26.4%	26	22.8%
	No	318	73.6%	91	28.6%
Availability of an accountant	Yes—facility with an accountant	393	91.0%	110	27.9%
	No	39	9.0%	7	17.9%
Availability of a noticeboard	Yes—facility with a noticeboard	403	93.3%	109	27.1%
	No	29	6.7%	8	27.6%
Availability of HFGC	Yes—facility with a HFGC	249	57.6%	73	29.3%
	No	183	42.4%	44	24.0%
Availability of health commodities	Average rating between 1–10 (SD)	432	6.6 (2.2)		
Facility infrastructure condition	Average rating between 1–10 (SD)	432	6.7 (2.3)		
Staffing level	Average rating between 1–10 (SD)	432	4.5 (2.1)		
Entitlements and benefits condition	Average rating between 1–10 (SD)	432	4.3 (2.5)		
Facility level of care	Hospital	188	43.5%	48	25.5%
	Health centre	244	56.5%	69	28.3%
Region	Dar es Salaam	194	44.9%	50	25.8%
	Pwani	238	55.1%	67	28.2%

*Notes*: HFGC, Health Facility Governing Committee; SD, standard deviation.

### Factors influencing informal payment

The results are presented in two models (Table 3), with model 1 showing results from mixed-effects model, while model 2 showing results from a standard logistic model with clustering at the facility level. As indicated in the analysis section, we had to estimate the logistic model (model 2) because the mixed-effects model (model 2) did not reflect improvement in model estimation. This was confirmed through a likelihood ratio test and ICC estimate. Consequently, the regression results from both models were almost similar, with few exceptions (Table 3). Our discussion of results, therefore, focused on the estimates from model 2 as the standard logistic model with clustering at the facility level.

**Table 3:** Logistic regression results

		Model 1 Mixed-effects model		Model 2 Logistic model	
Variables	Description	AOR	(95% CI)	AOR	(95% CI)
Panel A: Individual factors					
Age	20–34 years	1.00		1.00	
	35–44 years	0.46	(0.25–0.86)**	0.46	(0.25–0.84)**
	45–60 years	0.44	(0.22–0.86)**	0.44	(0.20–0.95)**
Gender	Male	0.72	(0.42–1.24)	0.72	(0.39–1.28)
	Female	1.00		1.00	
Medical cadre	Medical specialist	2.60	(0.99–6.82)*	2.60	(0.89–7.55)*
	Medical officer and Clinical officer	1.95	(0.98–3.89)*	1.93	(0.73–5.13)
	Nurse and Midwives	1.52	(0.79–2.94)	1.50	(0.71–3.18)
	Others (e.g. paramedics)	1.00		1.00	
Marital status	Married	1.21	(0.70–2.08)	1.20	(0.67–2.16)
	Not married	1.00		1.00	
Position at the facility level	In-charge of the department/unit	1.69	(1.02–2.81)**	1.72	(1.15–2.57)***
	Not in-charge	1.00		1.00	
Experience at the facility level	Number of years at a facility	1.00	(0.96–1.04)	1.00	(0.97–1.03)
Supplementary job	Has any supplementary job to earn income	1.44	(0.87–2.37)	1.43	(0.89–2.31)
	No supplementary job	1.00		1.00	
Panel B: Facility factors					
Supervision throughout	Facility with supervision throughout	0.43	(0.26–0.73)***	0.43	(0.26–0.70)***
	No	1.00		1.00	
Electronic mode of payment	Yes—facility with electronic payment	0.77	(0.40–1.47)	0.77	(0.38–1.57)
	No	1.00		1.00	
Availability of an accountant	Yes—facility with an accountant	1.90	(0.74–4.92)	1.91	(0.53–6.88)
	No	1.00		1.00	
Availability of a noticeboard	Yes—facility with a noticeboard	0.89	(0.34–2.35)	0.89	(0.38–2.07)
	No	1.00		1.00	
Availability of HFGC	Yes—facility with a HFGC	1.10	(0.66–1.85)	1.12	(0.62–2.04)
	No	1.00		1.00	
Availability of health commodities	Average rating between 1–10	0.98	(0.86–1.11)	0.98	(0.88–1.09)
Facility infrastructure condition	Average rating between 1–10	1.05	(0.93–1.17)	1.05	(0.93–1.17)
Staffing level	Average rating between 1–10	1.09	(0.97–1.24)	1.09	(0.98–1.22)
Entitlements and benefits condition	Average rating between 1–10	0.85	(0.76–0.96)***	0.85	(0.76–0.95)***
Facility level of care	Hospital	1.00		1.00	
	Health centre	1.05	(0.62–1.78)	1.05	(0.65–1.69)
Region	Pwani	1.00		1.00	
	Dar es Salaam	1.13	(0.64–1.97)	1.12	(0.60–2.09)
Number of observation (*n*)		432		432	
SD of random intercept		0.228			
*P*-value LR test vs logistic model		0.373			
Log likelihood		–228.1		–228.1	

*Notes*: AOR, adjusted odds ratio; CI, confidence interval; SD, standard deviation; HFGC, Health Facility Governing Committee; Model 1 is for mixed-effects model while Model 2 is for standard logistic regression model; *** denotes significance at 1%, **at 5% and *at 10% level.

The results from model 2 show that the likelihood of engaging in informal payment was significantly associated with health workers’ age and cadre (Table 3). Specifically, health workers aged between 35 and 44 years and between 45 and 60 years were less likely to engage in informal payment with odds ratios of [AOR = 0.46 (confidence interval (CI): 0.25–0.84)] and [AOR = 0.44 (CI: 0.20–0.95)], respectively, compared to younger health workers aged between 20 and 34 years. Moreover, the probability of engaging in informal payment was significantly higher among specialists [AOR = 2.60 (CI: 0.89–7.55)] compared to low-level cadres/seniority (e.g. paramedic, pharmacist, laboratory technicians, etc.). In other words, being a specialist increased the chances of engaging in informal payment by almost three times (and by two times for medical doctors), compared to other providers (e.g. paramedics) (Table 3). Moreover, being in charge of the department increased the likelihood of engaging in informal payment significantly [AOR = 1.72 (CI: 1.15–2.57)] (Table 3). Among facility-level factors, receiving informal payments was less likely among those with supervision throughout [AOR = 0.43 (CI: 0.26–0.70)]. Furthermore, higher perceived ratings on provision of entitlements and benefits significantly reduced the probability of engaging in informal payment (Table 3). Specifically, a single point increase in this rating significantly reduced the chances of informal payment by a factor of 0.85 (95% CI: 0.76–0.95).

We further sought to examine the effects on subgroup within provider, in relation to identified significant factors in Table 3 (e.g. supervision throughout and timely provision of entitlements and benefits at facility) that reduced the chances of engaging in informal payments. Subgroup effects showed that the effect of supervision was much stronger and significant among the younger and older staff than middle age staff; among medical officers/clinicians and nurses/midwives than specialist and paramedics; it did not vary in terms of staff position at the facility or whether a staff member has a supplementary job (Table 4, column 1). It had no effect on the specialists who were most likely to engage in informal payments. In terms of entitlements and benefits provision, the effect was significantly stronger in older compared to the younger providers; in medical officers/clinicians and lower cadres (e.g. paramedics) than specialist and nurses/midwives; and in those with no supplementary job (Table 4, column 2). Timely payment of entitlements and benefits was associated with fewer informal payments among older providers and certain cadres (medical officers and paramedics), and particularly among those with no additional job.

## Discussion

Our study identified supply-side factors influencing the practice of informal payments in Tanzania. We found that 27% out of 432 health workers engaged in informal payment, and the practice was common especially amongst younger (<35 years) and more senior (e.g. specialists) health workers, as well as heads of departments. We also found that good provision of entitlements and benefits and continued supervision was associated with less informal payment.

According to our first hypothesis, while 27% health workers ever engaged in informal payment, this varied by health workers’ characteristics. For instance, the age of health workers significantly affected their likelihood of engaging in informal payment. Specifically, younger health workers (<35 years) were more likely to engage in informal payment than older ones. This could reflect how younger health workers are more likely to have lower salaries than their older or more senior colleagues, so that informal payment is a compensating mechanism.

In terms of providers’ cadre and position, being more senior (e.g. specialists) was significantly associated with a higher likelihood of engaging in informal payment, as was a leadership role at the facility. This may reflect how senior staff and specialists are better able to solicit informal charges as they offer more specialized services, i.e. have more opportunities to engage in informal payments. A provider with higher professional status may be able to negotiate with patients from a position of authority. This echoes findings from Tajikistan that doctors were more likely to seek informal payment than lower staff in the hierarchy such as nurses, attendants and administrative staff (Dabalen and Wane, 2008). Similarly, a study by Miller *et al.* (2000) in four countries (Ukraine, Bulgaria, Slovakia and the Czech Republic) found that doctors received money and expensive presents while nurses received flowers, chocolate, and similar gifts of low value. Informal payments were also unequally distributed among health workers in Hungary (Gaal *et al.*, 2006b) and Bulgaria (Balabanova and McKee, 2002), with a larger share of the money going to family doctors and specialists. Other scholars found informal payments were more prevalent in the emergency departments or hospital units providing surgical services (Jafari *et al.*, 2015; Khodamoradi *et al.*, 2018), which is consistent with our finding because most specialists and senior doctors work in emergency or surgical departments.

We did not find a significant association between health workers’ gender and informal payment, even though more specialists were male (61%) and more heads of units were female (56%). The lack of a significant association with gender is consistent with some studies (e.g. Dabalen and Wane, 2008) but not others (e.g. Swamy *et al.*, 2001; Duflo and Topalova, 2004). We also found no significant association between informal payments and health workers’ marital status or facility type (e.g. level of care). Other studies have reported an association with facility type (in terms of ownership) (Kankeu *et al.*, 2016; Khodamoradi *et al.*, 2018) but, overall, evidence of an association between informal payments and facility ownership remains inconclusive in the literature.

The evidence on our second hypothesis is mixed. Frequently recommended oversight mechanisms, such as electronic payment, an accountant to check for fraud, and HFGCs were not significant. Nor were measures encouraging transparency, such as a noticeboard publicizing rules, services offered and rates of user fees. The evidence on HFGC remains mixed, with some studies suggesting that they can stimulate bottom-up accountability and possibly reduce informal payments (Gatti *et al.*, 2003; Rispel *et al.*, 2016), while others are less positive.

Higher levels of supervision and oversight were, however, significantly associated with lower levels of informal payment. This is intuitive and consistent with other evidence that supportive supervision and oversight facilitate implementation of any intervention. For instance, the implementation of the pro-poor exemption policy in Tanzania was more successful in districts with high commitment by health managers and local government officials, and in districts with regular supervisory visits (Maluka, 2013). Thus, regular supervision visits and oversight at the facility level, especially of heads of units and senior specialists, may act as a disincentive to health workers asking for informal payments. Importantly, supervision did not deter everyone equally. For example, it was not associated with fewer informal payments among senior specialists, while having a formal management role did not make a difference. This may be because specialists have higher status, stronger informal connections, and thus can avoid penalties. The role of informal relationships and networks needs further study, e.g. asking providers about their relationship with their supervisors.

It is, of course, possible that informal payments simply reflect low pay of health workers, especially for younger staff, with lower salaries and family responsibilities. Thus, timely provision of entitlements and benefits was associated with lower levels of informal payments. This was particularly the case for those with no supplementary job to augment their income. Low income and inadequate entitlements and benefits in most cases reflect inadequate health sector funding, so health workers may see informal payments as a coping mechanism (survival corruption) (Belita *et al.*, 2013). Our findings on timely provision of entitlements and benefits are consistent with evidence from Cameroon (Kankeu *et al.*, 2016) and Tajikistan (Dabalen and Wane, 2008). The likelihood of engaging in informal payment was higher in facilities where health workers received inadequate remuneration (Kankeu *et al.*, 2016), and among health workers receiving below their perceived fair-wage in Tajikistan (Dabalen and Wane, 2008). Other studies have also identified low salaries as important determinants of informal payment (Balabanova and McKee, 2002; Belli and Shahriari, 2002; Vian *et al.*, 2006; Jahangiri and Aryankhesal, 2017).

Informal payments can have many unwanted consequences ranging from impoverishment, inability to access care which is often of poor quality, low satisfaction with care and mistrust (Bardhan, 1997; Azfar *et al.*, 2001; Cho and Kirwin, 2007; Lewis, 2007; Habibov, 2016). Corruption can also undermine progress to improved service delivery by eroding confidence in public institutions (Clausen *et al.*, 2011), potentially creating vicious circles (Cho and Kirwin, 2007). Other scholars have proposed that corruption can strengthen trust if bribes enable access to otherwise inaccessible services (Méon and Weill, 2010), but this is contested (Lavallée *et al.*, 2008).

Our study has several policy implications. First, since the chances of engaging in informal payment varied by type of provider, we need to clarify who benefits from informal payments and who can be influenced through targeted incentives or penalties. Second, we must move beyond commonly recommended approaches involving accountability and transparency measures that may have limited impact and can be bypassed. However, other aspects of oversight, like supervision remain important; although evidence is being mixed, supportive supervision is anyway important for human resource management but it deters only some people from engaging in corrupt practices. Our theoretical model suggests a need for measures, adopting innovative strategies adapted to context. Third, there should be adequate health funding to ensure sufficient pay, allowances, and working conditions. This can be expected to reduce informal payments, but only as part of an overall strategy. Fourth, our findings highlight the importance of stronger accountability mechanisms, with enhanced monitoring and supervision of staff. Weak supervision and oversight, as observed in many LMICs, creates opportunities for rent seeking (Fjeldstad, 2004; SIKIKA, 2014). This calls for stronger accountability and responsiveness to service users. In the absence of adequate measures, the proliferation of an informal healthcare market, including informal payments, can undermine other measures to improve healthcare financing and delivery (Ensor and Witter, 2001). Further research is also needed to understand how to prevent some health workers from gaming the system, negotiating deals with supervisors, or using personal networks to bypass sanctions.

This study has some limitations. First, our definition of informal payment is narrow, capturing only the reported likelihood of engaging in it rather than actual amounts paid and payment frequency. However, for our purposes, this seemed preferable given the potential for misreporting of detailed information, difficulties of recall and sensitivity of the topic. Since engaging in informal payments was self-reported and this is sensitive, we placed these questions towards the end of the questionnaire, by when we had built a good rapport with respondents. Second, since our analysis relied on data from public hospitals and health centres and excluded dispensaries, we do not capture the entire range of informal payments across facility types. However, this decision was based on prior interviews and workshops which revealed that informal payments are less common in dispensaries, especially in rural areas, due to the greater poverty in the area, while their low staffing levels would have hampered implementation of the survey. Third, we did not examine informal mechanisms emerging among networks of providers. Political science literature suggests that those networks can be important but we will explore them in further analysis of our qualitative data. Lastly, since our question on whether a health provider ever received/asked informal payments is sensitive and personal, the face-to-face interview might induce information bias. However, we invested extensively in training data collectors to build a trusted rapport with respondents (including role play), and our consent form stated that the data will be anonymized and only used by researchers in the project.

## Conclusion

This study highlights how supply-side factors can influence informal payments in developing countries as stipulated in our theoretical framework. We found evidence of the hypotheses arising from the ACE approach, inspired by political settlement theory. Vertical enforcement of formal rules and more transparency might be not sufficient to reduce informal payments, especially when powerful players can bypass them easily. Conventional measures aimed at reducing informal payments might lead to different responses among the various actors, in some cases reinforcing underlying power distribution. Indeed, we found that the chances of engaging in informal payments varied by identifiable characteristics. This highlights the need to identify subgroups for targeted measures. We also showed the potential for providing entitlements and benefits in a timely way and of continued supervision in reducing informal payments. To this end, policy responses should address both different individual incentives and system-level factors, including ensuring adequate health sector financing, better and timely remuneration of frontline public health providers, and enhanced governance and supervision.
